# 4,4′-[2,2-Dimethyl­propane-1,3-diyl­bis(nitrilo­methyl­idyne)]dibenzonitrile

**DOI:** 10.1107/S1600536808018345

**Published:** 2008-06-19

**Authors:** Hoong-Kun Fun, Hadi Kargar, Reza Kia

**Affiliations:** a X-ray Crystallography Unit, School of Physics, Universiti Sains Malaysia, 11800 USM, Penang, Malaysia; b Department of Chemistry, School of Science, Payame Noor University (PNU), Ardakan, Yazd, Iran

## Abstract

The title compound, C_21_H_20_N_4_, is a bidentate Schiff base ligand. An intra­molecular C—H⋯N hydrogen bond forms a five-membered ring, producing an *S*(5) ring motif. The cyano and imino [–C(H_2_)—N=C–] functional groups are coplanar with the benzene ring in each half of the mol­ecule. The packing of the mol­ecules is controlled by C—H⋯π and π–π inter­actions [centroid-to-centroid distance = 3.6944 (8) Å].

## Related literature

For related literature on hydrogen-bond motifs, see Bernstein *et al.* (1995 ▶). For values of bond lengths, see Allen *et al.* (1987 ▶). For related structures, see, for example: Li *et al.* (2005 ▶); Bomfim *et al.* (2005 ▶); Glidewell *et al.* (2005 ▶, 2006 ▶); Sun *et al.* (2004 ▶); Habibi *et al.* (2007 ▶); Fun *et al.* (2008 ▶).
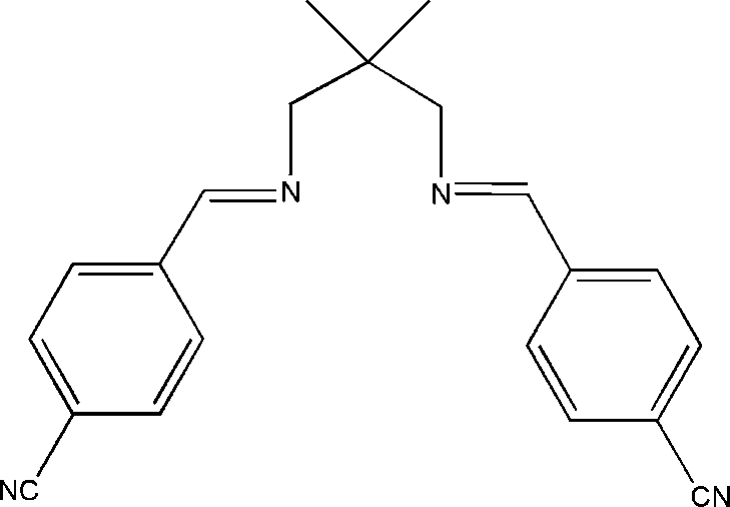


## Experimental

### 

#### Crystal data

C_21_H_20_N_4_
*M* *_r_* = 328.41
Monoclinic, 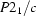

*a* = 6.3833 (2) Å
*b* = 34.0679 (8) Å
*c* = 8.5190 (2) Å
β = 100.654 (2)°
*V* = 1820.65 (8) Å^3^
*Z* = 4
Mo *K*α radiation
μ = 0.07 mm^−1^
*T* = 100.0 (1) K
0.42 × 0.20 × 0.19 mm

#### Data collection

Bruker SMART APEXII CCD area-detector diffractometer
Absorption correction: multi-scan (*SADABS*; Bruker 2005 ▶) *T*_min_ = 0.970, *T*_max_ = 0.986
21813 measured reflections
5329 independent reflections
3711 reflections with *I* > 2σ(*I*)
*R*_int_ = 0.046

#### Refinement

*R*[*F* ^2^ > 2σ(*F* ^2^)] = 0.056
*wR*(*F* ^2^) = 0.139
*S* = 1.04
5329 reflections
228 parameters
H-atom parameters constrained
Δρ_max_ = 0.24 e Å^−3^
Δρ_min_ = −0.20 e Å^−3^

### 

Data collection: *APEX2* (Bruker, 2005 ▶); cell refinement: *APEX2*; data reduction: *SAINT* (Bruker, 2005 ▶); program(s) used to solve structure: *SHELXTL* (Sheldrick, 2008 ▶); program(s) used to refine structure: *SHELXTL*; molecular graphics: *SHELXTL*; software used to prepare material for publication: *SHELXTL* and *PLATON* (Spek, 2003 ▶).

## Supplementary Material

Crystal structure: contains datablocks global, I. DOI: 10.1107/S1600536808018345/at2578sup1.cif

Structure factors: contains datablocks I. DOI: 10.1107/S1600536808018345/at2578Isup2.hkl

Additional supplementary materials:  crystallographic information; 3D view; checkCIF report

## Figures and Tables

**Table 1:** Hydrogen-bond geometry (Å, °) *Cg*1 is the centroid of the C12–C17 benzene ring.

*D*—H⋯*A*	*D*—H	H⋯*A*	*D*⋯*A*	*D*—H⋯*A*
C20—H20*B*⋯N2	0.96	2.57	2.923 (2)	102
C21—H21*C*⋯*Cg*1^i^	0.96	2.95	3.7193 (16)	138

Symmetry code: (i) 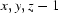.

**Fig. 1.**
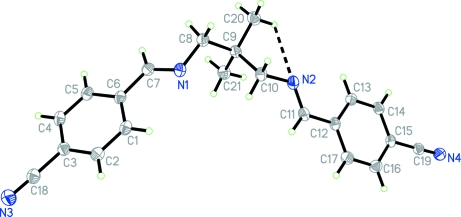
The molecular structure of the title compound with atom labels and 50% probability ellipsoids for non-H atoms. Intramolecular hydrogen bond is shown as a dashed line.

**Fig. 2.**
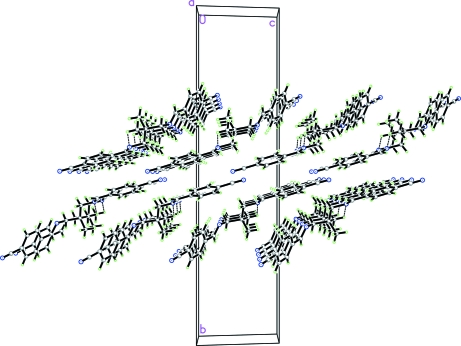
The crystal packing, viewed down the a axis, showing stacking of the benzene rings. Intramolecular H bonds are drawn as dashed lines.

## supplementary crystallographic information

### Comment

Schiff bases are one of most prevalent mixed-donor ligands in the field of coordination chemistry. They play an important role in the development of coordination chemistry related to catalysis and enzymatic reactions, magnetism, and supramolecular architectures. Structures of Schiff bases derived from substituted benzaldehydes and closely related to the title compound have been reported (Li *et al.*, 2005; Bomfim *et al.*, 2005; Glidewell *et al.*, 2005, 2006; Sun *et al.*, 2004; Habibi *et al.*, 2007; Fun *et al.*, 2008).

An intramolecular C—H···N hydrogen bond forms a five-membered ring, producing an *S*(5) ring motif (Bernstein *et al.*, 1995). The bond lengths and angles in the molecule (Fig. 1) are within normal ranges (Allen *et al.*, 1987). The cyano and imino (—C(H_2_)—N═C—) functional groups are coplanar with the benzene ring in each half of the molcule. The torsion angles of C6—C7—N1—C8 and C12—C11—N2—C10 are -178.95 (13) and -179.43 (12)°, respectively. The packing of the molecule, (Fig. 2), is controlled by C—H···π and π–π interactions [centroid-centroid distance being 3.6944 (8) Å] (Table 1).

### Experimental

The synthetic method has been described earlier (Fun *et al.*, 2008). Single crystals suitable for X-ray diffraction were obtained by evaporation of an ethanol solution at room temperature.

### Refinement

H atoms were positioned geometrically and refined using a riding model with C—H = 0.93 Å for aromatic H and 0.97 Å for methylene and 0.96 Å for methyl H atoms. The *U*_iso_ values were constrained to be 1.5*U*_eq_ of the carrier atom for the methyl H atoms and 1.2*U*_eq_ for the remaining H atoms. A rotating-group model was used for the methyl groups.

### Crystal data

**Table:**

C_21_H_20_N_4_	*F*_000_ = 696
*M**_r_* = 328.41	*D*_x_ = 1.198 Mg m^−^^3^
Monoclinic, *P*2_1_/*c*	Mo *K*α radiation λ = 0.71073 Å
Hall symbol: -P 2ybc	Cell parameters from 3870 reflections
*a* = 6.3833 (2) Å	θ = 2.4–29.3º
*b* = 34.0679 (8) Å	µ = 0.07 mm^−^^1^
*c* = 8.5190 (2) Å	*T* = 100.0 (1) K
β = 100.654 (2)º	Block, colourless
*V* = 1820.65 (8) Å^3^	0.42 × 0.20 × 0.19 mm
*Z* = 4	

### Data collection

**Table:**

Bruker SMART APEXII CCD area-detector diffractometer	5329 independent reflections
Radiation source: fine-focus sealed tube	3711 reflections with *I* > 2σ(*I*)
Monochromator: graphite	*R*_int_ = 0.046
*T* = 100.0(1) K	θ_max_ = 30.2º
φ and ω scans	θ_min_ = 1.2º
Absorption correction: multi-scan(SADABS; Bruker 2005)	*h* = −9→7
*T*_min_ = 0.970, *T*_max_ = 0.986	*k* = −48→48
21813 measured reflections	*l* = −12→12

### Refinement

**Table:**

Refinement on *F*^2^	Secondary atom site location: difference Fourier map
Least-squares matrix: full	Hydrogen site location: inferred from neighbouring sites
*R*[*F*^2^ > 2σ(*F*^2^)] = 0.056	H-atom parameters constrained
*wR*(*F*^2^) = 0.139	*w* = 1/[σ^2^(*F*_o_^2^) + (0.0568*P*)^2^ + 0.5007*P*] where *P* = (*F*_o_^2^ + 2*F*_c_^2^)/3
*S* = 1.04	(Δ/σ)_max_ < 0.001
5329 reflections	Δρ_max_ = 0.24 e Å^−^^3^
228 parameters	Δρ_min_ = −0.19 e Å^−^^3^
Primary atom site location: structure-invariant direct methods	Extinction correction: none

### Special details

**Table:**

Experimental. The low-temperature data was collected with the Oxford Cyrosystem Cobra low-temperature attachment.
Geometry. All e.s.d.'s (except the e.s.d. in the dihedral angle between two l.s. planes) are estimated using the full covariance matrix. The cell e.s.d.'s are taken into account individually in the estimation of e.s.d.'s in distances, angles and torsion angles; correlations between e.s.d.'s in cell parameters are only used when they are defined by crystal symmetry. An approximate (isotropic) treatment of cell e.s.d.'s is used for estimating e.s.d.'s involving l.s. planes.
Refinement. Refinement of *F*^2^ against ALL reflections. The weighted *R*-factor *wR* and goodness of fit *S* are based on *F*^2^, conventional *R*-factors *R* are based on *F*, with *F* set to zero for negative *F*^2^. The threshold expression of *F*^2^ > σ(*F*^2^) is used only for calculating *R*-factors(gt) *etc*. and is not relevant to the choice of reflections for refinement. *R*-factors based on *F*^2^ are statistically about twice as large as those based on *F*, and *R*- factors based on ALL data will be even larger.

### Fractional atomic coordinates and isotropic or equivalent isotropic displacement parameters (Å^2^)

**Table:**

	*x*	*y*	*z*	*U*_iso_*/*U*_eq_	
N1	0.8180 (2)	0.14310 (4)	1.22220 (14)	0.0240 (3)	
N2	0.7297 (2)	0.07939 (4)	0.76666 (14)	0.0243 (3)	
N3	0.1075 (3)	0.23585 (4)	1.75196 (17)	0.0346 (4)	
N4	0.2208 (2)	0.01242 (4)	−0.06341 (15)	0.0278 (3)	
C1	0.4959 (3)	0.16261 (4)	1.41380 (17)	0.0234 (3)	
H1A	0.4844	0.1376	1.3689	0.028*	
C2	0.3530 (3)	0.17457 (4)	1.50763 (17)	0.0243 (3)	
H2A	0.2462	0.1576	1.5267	0.029*	
C3	0.3698 (3)	0.21224 (4)	1.57374 (16)	0.0226 (3)	
C4	0.5288 (3)	0.23767 (4)	1.54525 (17)	0.0254 (3)	
H4A	0.5386	0.2628	1.5886	0.031*	
C5	0.6719 (3)	0.22545 (4)	1.45229 (17)	0.0237 (3)	
H5A	0.7789	0.2424	1.4335	0.028*	
C6	0.6573 (3)	0.18785 (4)	1.38618 (16)	0.0211 (3)	
C7	0.8171 (3)	0.17624 (4)	1.29000 (16)	0.0224 (3)	
H7A	0.9230	0.1942	1.2785	0.027*	
C8	0.9911 (3)	0.13680 (4)	1.13468 (17)	0.0245 (3)	
H8A	1.0761	0.1147	1.1816	0.029*	
H8B	1.0823	0.1598	1.1469	0.029*	
C9	0.9149 (3)	0.12886 (4)	0.95573 (16)	0.0215 (3)	
C10	0.7881 (3)	0.09017 (4)	0.93463 (16)	0.0232 (3)	
H10A	0.8736	0.0694	0.9923	0.028*	
H10B	0.6600	0.0930	0.9796	0.028*	
C11	0.5405 (3)	0.06825 (4)	0.71580 (16)	0.0226 (3)	
H11A	0.4445	0.0675	0.7857	0.027*	
C12	0.4686 (3)	0.05644 (4)	0.54744 (16)	0.0212 (3)	
C13	0.6067 (3)	0.05915 (4)	0.43861 (17)	0.0231 (3)	
H13A	0.7442	0.0687	0.4719	0.028*	
C14	0.5402 (3)	0.04776 (4)	0.28203 (17)	0.0233 (3)	
H14A	0.6322	0.0497	0.2096	0.028*	
C15	0.3345 (3)	0.03329 (4)	0.23295 (16)	0.0212 (3)	
C16	0.1937 (3)	0.03068 (4)	0.33940 (17)	0.0256 (3)	
H16A	0.0561	0.0212	0.3058	0.031*	
C17	0.2624 (3)	0.04247 (4)	0.49654 (17)	0.0255 (3)	
H17A	0.1694	0.0410	0.5685	0.031*	
C18	0.2220 (3)	0.22512 (4)	1.67256 (18)	0.0270 (3)	
C19	0.2687 (3)	0.02115 (4)	0.06860 (17)	0.0227 (3)	
C20	1.1140 (3)	0.12547 (5)	0.88075 (19)	0.0287 (4)	
H20A	1.1895	0.1500	0.8920	0.043*	
H20B	1.0729	0.1192	0.7695	0.043*	
H20C	1.2046	0.1051	0.9337	0.043*	
C21	0.7745 (3)	0.16266 (4)	0.87814 (18)	0.0274 (4)	
H21A	0.8507	0.1870	0.8986	0.041*	
H21B	0.6466	0.1638	0.9222	0.041*	
H21C	0.7381	0.1584	0.7650	0.041*	

### Atomic displacement parameters (Å^2^)

**Table:**

	*U*^11^	*U*^22^	*U*^33^	*U*^12^	*U*^13^	*U*^23^
N1	0.0265 (8)	0.0254 (6)	0.0199 (6)	−0.0005 (5)	0.0037 (5)	−0.0025 (5)
N2	0.0282 (8)	0.0233 (6)	0.0222 (6)	−0.0009 (5)	0.0070 (5)	−0.0030 (5)
N3	0.0335 (10)	0.0336 (8)	0.0379 (8)	0.0006 (6)	0.0099 (7)	−0.0063 (6)
N4	0.0268 (8)	0.0305 (7)	0.0270 (6)	−0.0013 (6)	0.0071 (6)	−0.0028 (5)
C1	0.0282 (9)	0.0186 (7)	0.0219 (7)	−0.0002 (6)	0.0011 (6)	−0.0015 (5)
C2	0.0257 (9)	0.0222 (7)	0.0244 (7)	−0.0010 (6)	0.0028 (6)	0.0011 (5)
C3	0.0230 (9)	0.0247 (7)	0.0193 (6)	0.0030 (6)	0.0015 (6)	−0.0003 (5)
C4	0.0319 (10)	0.0203 (7)	0.0234 (7)	0.0002 (6)	0.0034 (6)	−0.0040 (5)
C5	0.0275 (9)	0.0212 (7)	0.0222 (7)	−0.0033 (6)	0.0037 (6)	−0.0006 (5)
C6	0.0236 (9)	0.0214 (7)	0.0168 (6)	0.0013 (6)	0.0002 (6)	0.0006 (5)
C7	0.0232 (9)	0.0225 (7)	0.0206 (6)	−0.0012 (6)	0.0019 (6)	0.0007 (5)
C8	0.0250 (9)	0.0235 (7)	0.0244 (7)	0.0015 (6)	0.0029 (6)	−0.0025 (5)
C9	0.0232 (9)	0.0200 (7)	0.0220 (6)	0.0002 (6)	0.0059 (6)	−0.0009 (5)
C10	0.0275 (9)	0.0234 (7)	0.0191 (6)	0.0005 (6)	0.0056 (6)	−0.0007 (5)
C11	0.0286 (10)	0.0193 (7)	0.0218 (7)	0.0010 (6)	0.0095 (6)	0.0000 (5)
C12	0.0277 (9)	0.0152 (6)	0.0214 (6)	0.0012 (6)	0.0063 (6)	0.0000 (5)
C13	0.0234 (9)	0.0226 (7)	0.0240 (7)	−0.0016 (6)	0.0058 (6)	−0.0023 (5)
C14	0.0269 (9)	0.0225 (7)	0.0226 (7)	−0.0003 (6)	0.0101 (6)	−0.0013 (5)
C15	0.0266 (9)	0.0161 (6)	0.0211 (6)	0.0020 (6)	0.0051 (6)	−0.0004 (5)
C16	0.0256 (9)	0.0258 (7)	0.0257 (7)	−0.0031 (6)	0.0054 (6)	−0.0004 (6)
C17	0.0274 (10)	0.0289 (8)	0.0227 (7)	−0.0025 (7)	0.0107 (6)	0.0002 (6)
C18	0.0300 (10)	0.0236 (7)	0.0268 (7)	0.0002 (7)	0.0037 (7)	−0.0020 (6)
C19	0.0240 (9)	0.0199 (7)	0.0253 (7)	0.0007 (6)	0.0075 (6)	0.0000 (5)
C20	0.0284 (10)	0.0290 (8)	0.0309 (8)	−0.0013 (7)	0.0112 (7)	−0.0014 (6)
C21	0.0306 (10)	0.0242 (7)	0.0274 (7)	0.0020 (7)	0.0053 (7)	0.0009 (6)

### Geometric parameters (Å, °)

**Table:**

N1—C7	1.2687 (18)	C9—C21	1.532 (2)
N1—C8	1.459 (2)	C9—C10	1.539 (2)
N2—C11	1.264 (2)	C10—H10A	0.9700
N2—C10	1.4576 (17)	C10—H10B	0.9700
N3—C18	1.143 (2)	C11—C12	1.4786 (19)
N4—C19	1.1486 (19)	C11—H11A	0.9300
C1—C2	1.380 (2)	C12—C17	1.391 (2)
C1—C6	1.395 (2)	C12—C13	1.396 (2)
C1—H1A	0.9300	C13—C14	1.379 (2)
C2—C3	1.398 (2)	C13—H13A	0.9300
C2—H2A	0.9300	C14—C15	1.392 (2)
C3—C4	1.389 (2)	C14—H14A	0.9300
C3—C18	1.444 (2)	C15—C16	1.392 (2)
C4—C5	1.379 (2)	C15—C19	1.446 (2)
C4—H4A	0.9300	C16—C17	1.389 (2)
C5—C6	1.396 (2)	C16—H16A	0.9300
C5—H5A	0.9300	C17—H17A	0.9300
C6—C7	1.476 (2)	C20—H20A	0.9600
C7—H7A	0.9300	C20—H20B	0.9600
C8—C9	1.536 (2)	C20—H20C	0.9600
C8—H8A	0.9700	C21—H21A	0.9600
C8—H8B	0.9700	C21—H21B	0.9600
C9—C20	1.529 (2)	C21—H21C	0.9600
			
C7—N1—C8	115.55 (14)	N2—C10—H10B	109.4
C11—N2—C10	117.85 (13)	C9—C10—H10B	109.4
C2—C1—C6	120.21 (13)	H10A—C10—H10B	108.0
C2—C1—H1A	119.9	N2—C11—C12	121.33 (14)
C6—C1—H1A	119.9	N2—C11—H11A	119.3
C1—C2—C3	119.66 (15)	C12—C11—H11A	119.3
C1—C2—H2A	120.2	C17—C12—C13	119.54 (13)
C3—C2—H2A	120.2	C17—C12—C11	120.10 (14)
C4—C3—C2	120.41 (14)	C13—C12—C11	120.36 (15)
C4—C3—C18	119.30 (14)	C14—C13—C12	120.32 (15)
C2—C3—C18	120.29 (15)	C14—C13—H13A	119.8
C5—C4—C3	119.66 (14)	C12—C13—H13A	119.8
C5—C4—H4A	120.2	C13—C14—C15	119.65 (14)
C3—C4—H4A	120.2	C13—C14—H14A	120.2
C4—C5—C6	120.45 (15)	C15—C14—H14A	120.2
C4—C5—H5A	119.8	C16—C15—C14	120.88 (13)
C6—C5—H5A	119.8	C16—C15—C19	120.37 (15)
C1—C6—C5	119.60 (14)	C14—C15—C19	118.75 (14)
C1—C6—C7	122.45 (13)	C17—C16—C15	118.87 (16)
C5—C6—C7	117.95 (14)	C17—C16—H16A	120.6
N1—C7—C6	123.57 (15)	C15—C16—H16A	120.6
N1—C7—H7A	118.2	C16—C17—C12	120.73 (14)
C6—C7—H7A	118.2	C16—C17—H17A	119.6
N1—C8—C9	113.72 (13)	C12—C17—H17A	119.6
N1—C8—H8A	108.8	N3—C18—C3	178.73 (19)
C9—C8—H8A	108.8	N4—C19—C15	177.74 (17)
N1—C8—H8B	108.8	C9—C20—H20A	109.5
C9—C8—H8B	108.8	C9—C20—H20B	109.5
H8A—C8—H8B	107.7	H20A—C20—H20B	109.5
C20—C9—C21	109.99 (12)	C9—C20—H20C	109.5
C20—C9—C8	107.02 (13)	H20A—C20—H20C	109.5
C21—C9—C8	110.40 (12)	H20B—C20—H20C	109.5
C20—C9—C10	110.25 (12)	C9—C21—H21A	109.5
C21—C9—C10	109.88 (13)	C9—C21—H21B	109.5
C8—C9—C10	109.26 (11)	H21A—C21—H21B	109.5
N2—C10—C9	111.35 (11)	C9—C21—H21C	109.5
N2—C10—H10A	109.4	H21A—C21—H21C	109.5
C9—C10—H10A	109.4	H21B—C21—H21C	109.5
			
C6—C1—C2—C3	0.6 (2)	C21—C9—C10—N2	64.21 (16)
C1—C2—C3—C4	0.2 (2)	C8—C9—C10—N2	−174.52 (13)
C1—C2—C3—C18	−179.80 (14)	C10—N2—C11—C12	−179.43 (12)
C2—C3—C4—C5	−0.6 (2)	N2—C11—C12—C17	176.85 (14)
C18—C3—C4—C5	179.36 (15)	N2—C11—C12—C13	−3.1 (2)
C3—C4—C5—C6	0.3 (2)	C17—C12—C13—C14	−0.6 (2)
C2—C1—C6—C5	−0.8 (2)	C11—C12—C13—C14	179.35 (13)
C2—C1—C6—C7	178.55 (14)	C12—C13—C14—C15	−0.4 (2)
C4—C5—C6—C1	0.4 (2)	C13—C14—C15—C16	0.9 (2)
C4—C5—C6—C7	−179.02 (14)	C13—C14—C15—C19	−179.39 (13)
C8—N1—C7—C6	−178.95 (13)	C14—C15—C16—C17	−0.5 (2)
C1—C6—C7—N1	1.7 (2)	C19—C15—C16—C17	179.79 (14)
C5—C6—C7—N1	−178.92 (14)	C15—C16—C17—C12	−0.4 (2)
C7—N1—C8—C9	−119.58 (14)	C13—C12—C17—C16	1.0 (2)
N1—C8—C9—C20	176.60 (12)	C11—C12—C17—C16	−178.95 (13)
N1—C8—C9—C21	56.91 (17)	C4—C3—C18—N3	−19 (9)
N1—C8—C9—C10	−64.04 (16)	C2—C3—C18—N3	161 (9)
C11—N2—C10—C9	−134.31 (14)	C16—C15—C19—N4	149 (5)
C20—C9—C10—N2	−57.17 (17)	C14—C15—C19—N4	−31 (5)

### Hydrogen-bond geometry (Å, °)

**Table:**

*D*—H···*A*	*D*—H	H···*A*	*D*···*A*	*D*—H···*A*
C20—H20B···N2	0.96	2.57	2.923 (2)	102
C21—H21C···Cg1^i^	0.96	2.95	3.7193 (16)	138

Symmetry codes: (i) *x*, *y*, *z*−1.
